# Transposon insertion mapping with PIMMS – Pragmatic Insertional Mutation Mapping System

**DOI:** 10.3389/fgene.2015.00139

**Published:** 2015-04-09

**Authors:** Adam M. Blanchard, James A. Leigh, Sharon A. Egan, Richard D. Emes

**Affiliations:** ^1^School of Veterinary Medicine and Science, University of NottinghamLoughborough, UK; ^2^Advanced Data Analysis Centre, University of NottinghamLoughborough, UK

**Keywords:** TnSeq, INseq, TraDIS, transposon mapping, sequencing

## Abstract

The PIMMS (Pragmatic Insertional Mutation Mapping System) pipeline has been developed for simple conditionally essential genome discovery experiments in bacteria. Capable of using raw sequence data files alongside a FASTA sequence of the reference genome and GFF file, PIMMS will generate a tabulated output of each coding sequence with corresponding mapped insertions accompanied with normalized results enabling streamlined analysis. This allows for a quick assay of the genome to identify conditionally essential genes on a standard desktop computer prioritizing results for further investigation.

Availability: The PIMMS script, manual and accompanying test data is freely available at https://github.com/ADAC-UoN/PIMMS

## Introduction

Identification of essential genes using random mutagenesis has been used in numerous bacteria to identify genes that are conditionally essential; making random mutagenesis mapping a valuable tool to couple microbial genotype with phenotype. With the advent of next generation sequencing, various approaches for mapping the essential genome have been developed such as INSeq (Goodman et al., 2009), Tn-Seq (van Opijnen et al., 2009), HITS (Gawronski et al., 2009), and TraDIS (Langridge et al., 2009). These methods are capable of producing vast amounts of data, however, their analysis can be a daunting task for those not familiar with bioinformatics or data management. In order to make this experimental approach more accessible to a wider audience, a complete analysis package is required which can be utilized by those with minimal bioinformatics knowledge. Currently, there are no commercial software packages available that can deal with the complex nature of the data generated from Tn-mapping. Web-based tools do exist such as ESSENTIALS (Zomer et al., 2012), however, such tools which retain data on-line may not be a suitable platform for all end-users. Pragmatic Insertional, Mutation Mapping System (PIMMS) has been developed to analyze sequencing data from any random mutagenesis experiment. PIMMS is written in the perl programming language and will work on unix based systems with only the addition of freely available tools: a standard sequence aligner, perl packages Getopt::Long and Statistics::Descriptive, fastx toolkit, and R for graphical visualizations.

## The PIMMS Pipeline

**FIGURE 1:**
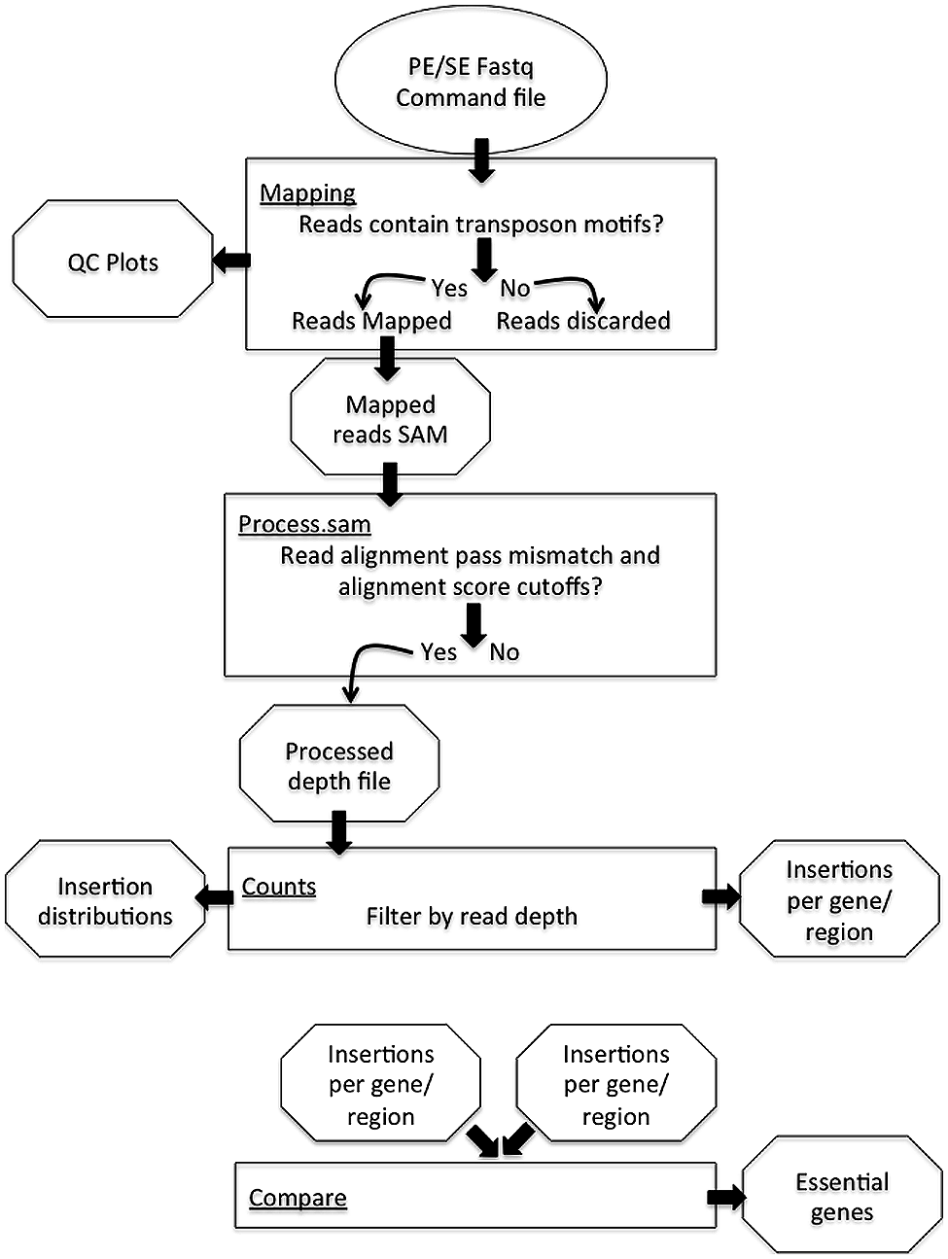
Schematic of the Pragmatic Insertional Mutation Mapping System (PIMMS) pipeline.

The PIMMS pipeline comprises four modules; mapping, process.sam, counts, and compare (**Figure 1A**). Examples shown are from data-sets resulting from experiments conducted as part of a PIMMS development project. Consequently, the results and biological interpretation of these are not discussed here but will be presented elsewhere (Blanchard et al., in preparation). For full description of parameter options and a description of output types and directory structure please see the PIMMS handbook available at .

### PIMMS Mapping

**FIGURE 2:**
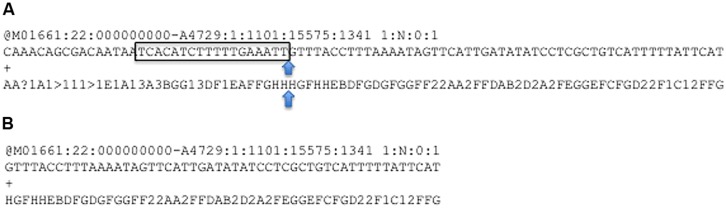
Process of read motif matching and trimming. **(A)** Reads in fastq format are scanned for motifs which are designated by the user to correspond to the end of the transposon element used in mutant library construction, matched motif shown as box. **(B)** The sequence following the motif, which will potentially include the bacterial genome sequence, is retained along with the corresponding phred quality scores.

A simple and universal approach for sample enrichment for Tn-mapping is through inverse PCR from the inserted element or transposon into flanking genomic DNA. The initial problem to overcome when dealing with these data is that the resulting sequence reads will consist of components from both transposon and chromosome, meaning that the raw sequence will not map faithfully to the target genome. Therefore, the first step is to separate these and create a file containing only the part of the read that follows from the end of the transposon fragment. Control of PIMMS mapping is primarily by the PIMMS.commands.txt file which must contain motifs (corresponding to the Tn termini) to be matched in the sequence reads and can additionally contain aligner commands (an example command.txt file is provided when downloaded from GitHub). The decision to have this commands file rather than ask for this information on the command line was to avoid potential typographical errors when entering the sequence motif on the command line. Sequence data entered into the PIMMS pipeline does not require any pre-processing, but rather uses raw fastq files. Reads that contain either of the motifs (including those on the reverse strand as reverse complement motifs are automatically generated) are identified and the sequence immediately following the motif and its corresponding quality score are extracted to a new fastq file (**Figure 2**). The user can specify a minimum and maximum length of potential genome sequence. As a default we retain sequences of minimum 20, maximum 50 bp. Keeping the maximum length relatively short avoids problems of re-entering the transposon where the PCR product was generated from a small circular template. To avoid double counting of insertion positions where both ends of a single insertion are sequenced, a single read is discarded if reads are from a paired end sequence experiment and extracted reads remain as paired after processing. If single end reads are used as input then this step is not considered. As a result, a combined single end read fastq file is aligned to the reference genome using the parameters provided in the PIMMS.command.txt file. The current version (version 1.9), allows automatic use of bowtie2 (Langmead and Salzberg, 2012) or BWA (Li and Durbin, 2009). Other aligners could also be used if given as a command in the PIMMS.command.txt. Any non-standard aligner will be queried by PIMMS prior to running. By default, BWA with options “mem –t 2” is used. BWA is recommended as this produced the most robust results in our test datasets. (See PIMMS process.sam for discussion). The resulting output is a mapped reads file in SAM format^1^ and plots of percent nucleotide distribution at each read position and read quality quartile plots of raw and processed reads (those that match a motif and post trimming).

### PIMMS process.sam

Whilst we generally use the PIMMS process.sam module to process reads directly following PIMMS mapping, the PIMMS pipeline can be initiated at this step following any read mapping that produces a standard SAM formatted output. Each read in the SAM file is assessed for number of mismatches between read and reference sequence (using the “MD” tag of the SAM file) and alignment score (using the “AS” tag of the SAM file). Reads with mismatches greater than that chosen by the user or alignment scores less than requested are ignored. For those that exceed user’s criteria the position of the initial base of the alignment (that immediately adjacent to the insertional element) is recorded. At this processing step, insertion positions can also be collapsed if they are exactly a given distance apart. This is important as the transposon system pGhost9::ISS1 used to develop this protocol incorporates an exact 8 bp repeat during insertion. The process.sam generates a simple text file of insertion coordinates and read depth at each unique insertion position in addition a log file of all parameter choices and run statistics is generated.

**FIGURE 3:**
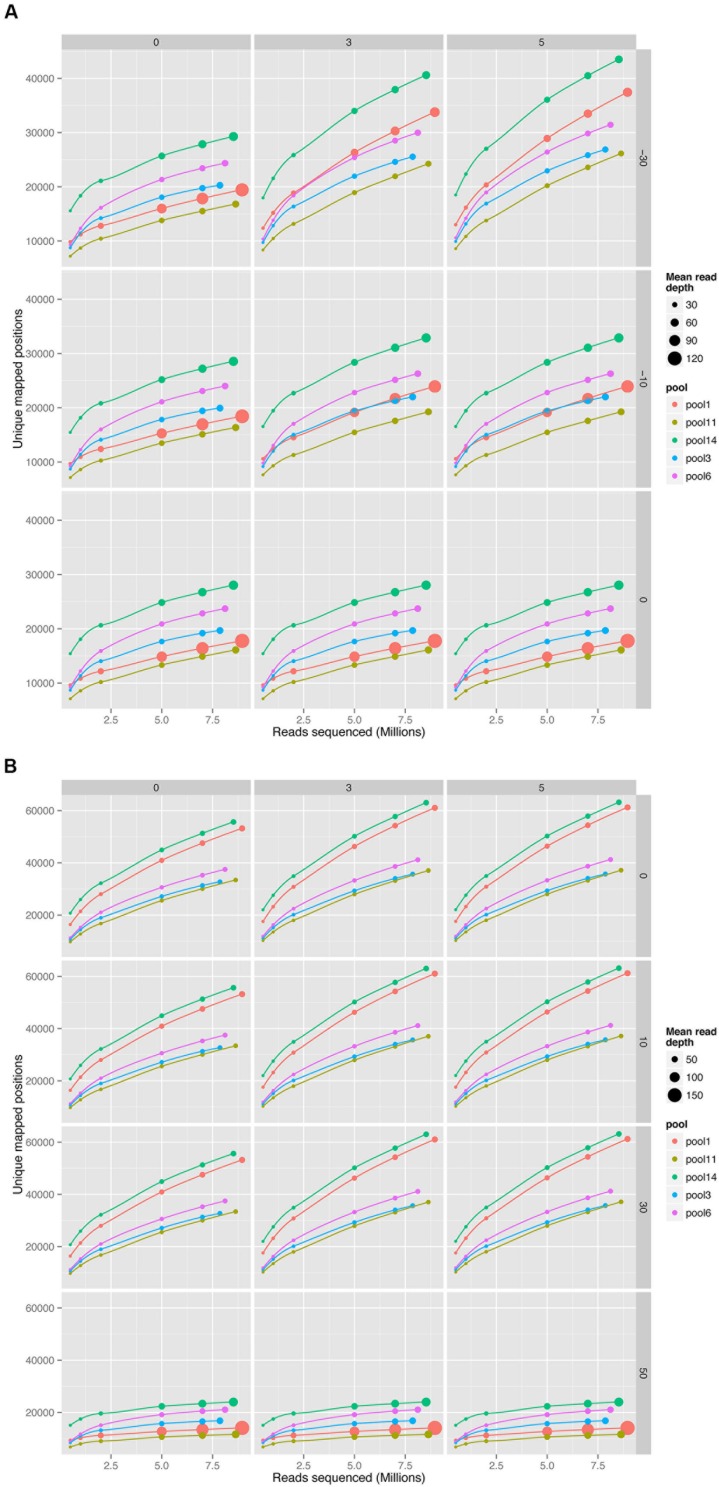
Comparison of read aligners, cumulative number of unique positions mapped from five pools of mutant *Streptococcus uberis* sampled at increasing sequence depth. **(A)** Bowtie2 (version 2.2.1) with parameters –end-to-end –very-sensitive. **(B)** BWA mem (version 0.7.10) with default parameters. Columns of the plot show changes with mismatches (0, 3, or 5 mismatches per read), and rows changes in alignment scores. Alignment scores were 0, 10, 30, and 50 for BWA and 0, -10, and -30 for Bowtie2. Whilst Bowtie2 uses negative numbers, for each aligner higher alignment scores represent higher confidence alignments (BWA = 50 and Bowtie2 = 0 are highest stringencies). Size of circles represents the mean number of reads mapping to each unique position with increasing read depth.

**FIGURE 4:**
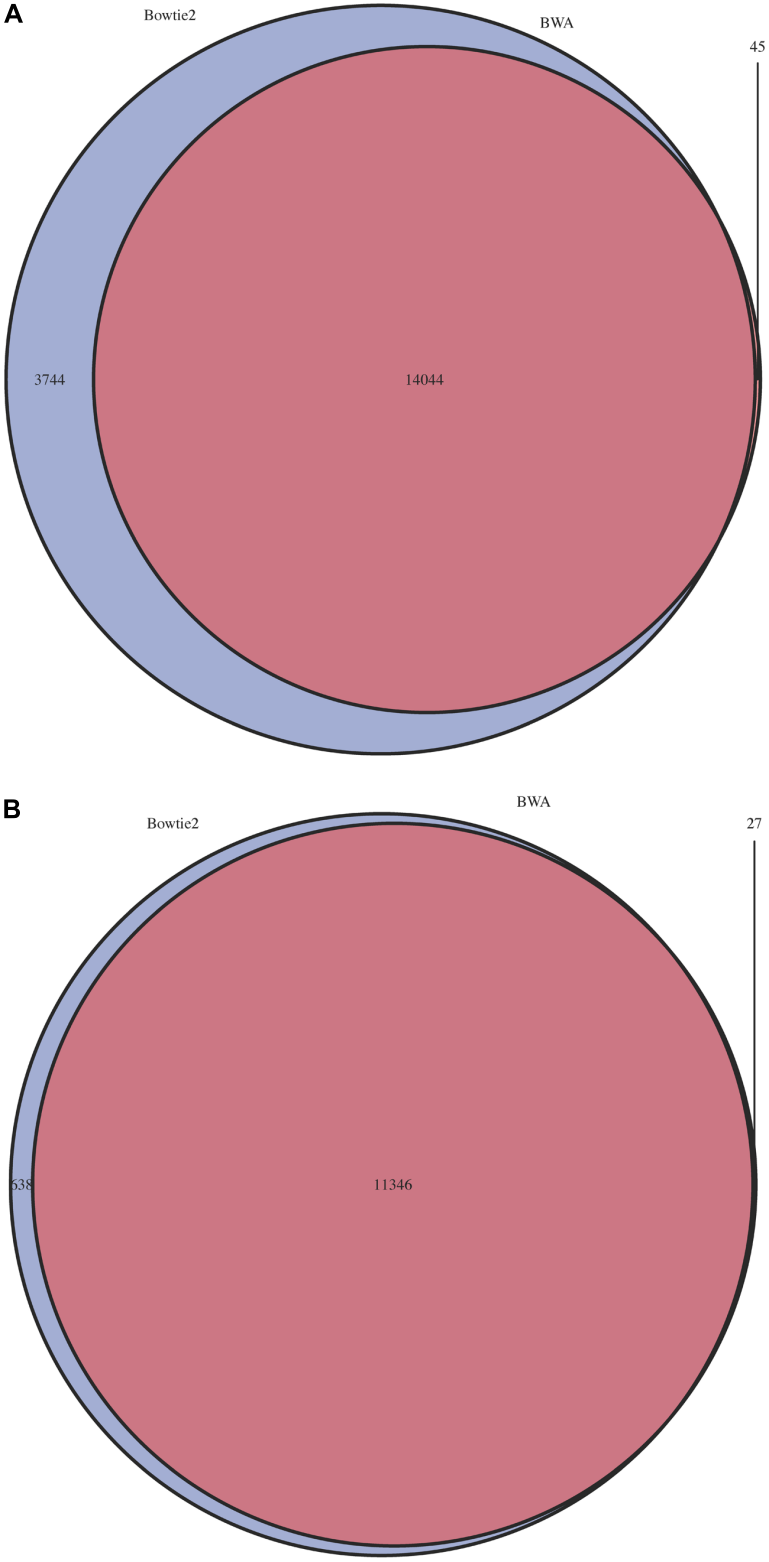
Overlap of predicted insertion positions between BWA and Bowtie2. For a single pool with an estimated number of possible mutations of 16,000 mutants estimated by colony counts, positions of predicted mutations were compared. Blue = Bowtie2, Purple = BWA. **(A)** All mutation positions. **(B)** Mutation positions with a minimum depth of >1.

The impact of choice of aligner and mismatch plus alignment score parameters was determined for five pools of mutant libraries generated in the bacterium *Streptococcus uberis* strain 0140J (ATCC BAA-854/0140J, Ward et al., 2009). Using the PIMMS mapping module BWA mem (version 0.7.10) with default parameters and Bowtie2 (version 2.2.1) with parameters –end-to-end –very-sensitive were used to map reads and results were assessed using PIMMS process.sam. For both aligners the mismatches allowed were 0, 3, and 5 (PIMMS.pl process.sam options –mis 0, 3, or 5). Whilst fixed integers (number of mismatched bases in a read) were used here, PIMMS.pl process.sam does allow for filtering on a maximum number of mismatches as a proportion of the read length for example –mis 0.1 allows 10% of bases for each read to mismatch (see PIMMS handbook or help). Alignment scores were 0, 10, 30, and 50 for BWA (PIMMS.pl process.sam options – a 0, 10, 30, and 50) and 0, -10, and -30 for Bowtie2 (PIMMS.pl process.sam options – a 0, neg10, and neg30). Whilst Bowtie2 uses negative numbers for low stringency for each aligner higher alignment scores represent higher confidence alignments. We observed that the alignment score has the dominant effect on both aligners. When this is set to the most stringent filter (BWA = 50, Bowtie2 = 0) the number of mismatches has little or no effect (**Figure 3**). This is likely due to the fact that alignments at this high stringency will have no mismatches by definition. Colonies were harvested from growth conditions selective for genomic integration of the mutagen and each was estimated to contain between 10,000 and 20,000 individuals. Due to the methods of production of such mutants the number of colonies is likely to correspond to a similar number of unique mutations within the pool, based on a single insertion event of the transposon occurring per bacterial colony harvested. Comparison of the low stringency setting with expected unique insertion detection identified multiple mismatching alignments, whilst at high stringency settings (mismatch = 0, align score = 50) BWA reproduced results within the range expected from original colony counts of library pools. In addition when the unique insertion positions were compared between aligners, BWA positions were almost entirely contained within predictions of Bowtie2 (**Figure 4**). The removal of positions with a single sequence read at a position dramatically reduces the Bowtie2 unique positions. Suggesting that the majority of disagreement was due to insertions with low read depth in Bowtie2. Whilst data for a single pool of mutants is shown, the same trend is seen across all experiments we have conducted. We therefore have engineered PIMMS to default to BWA aligner and we recommend use of filters alignment score = 50 and mismatch = 0, (PIMMS –m process.sam –a 50 –mis 0). We also recommend filtering of positions where only a single read is mapped. This is achieved with the coverage (-cov) option of PIMMS counts module (see PIMMS counts PIMMS Counts below).

### PIMMS Counts

**FIGURE 5:**
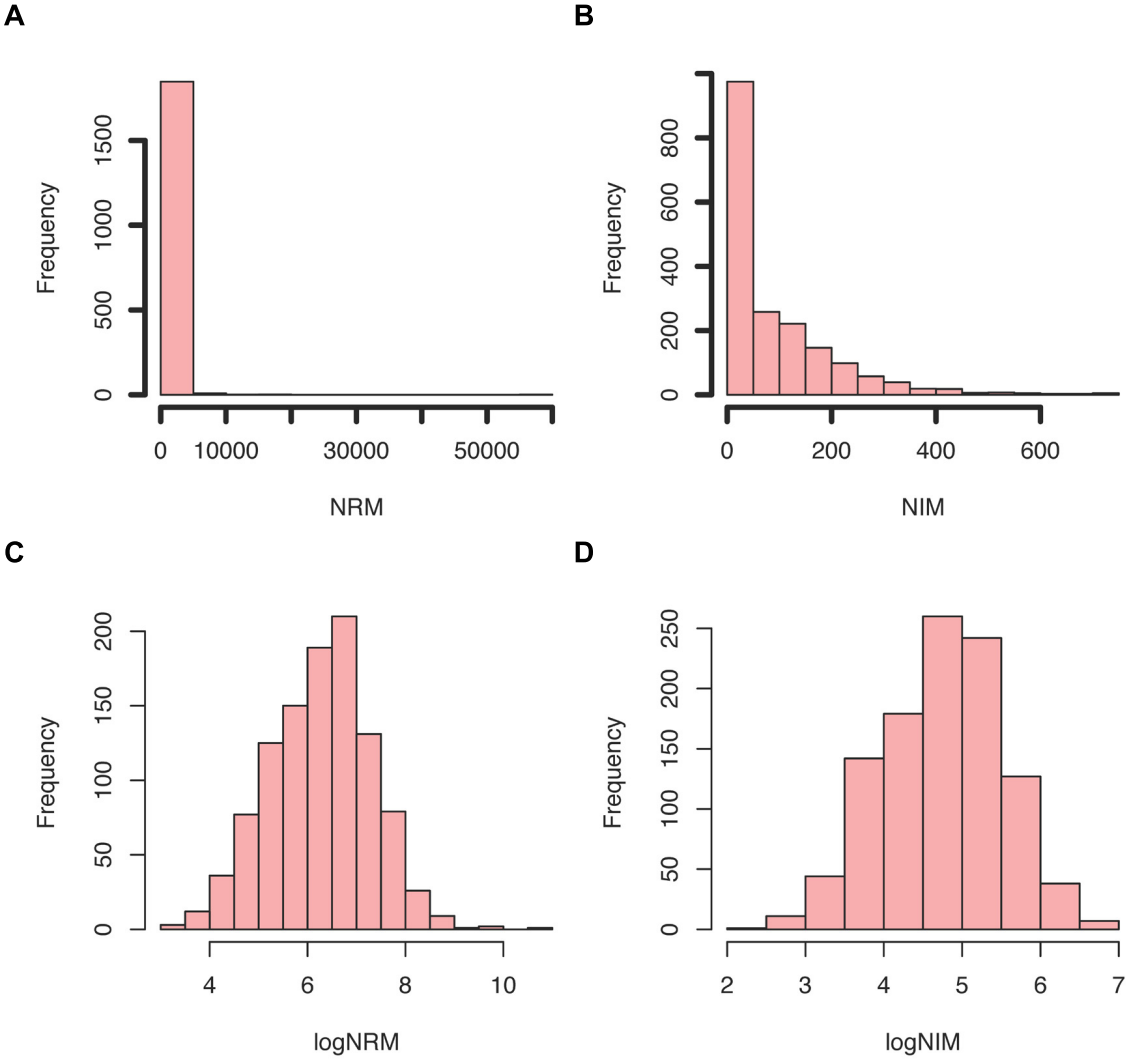
Example summary plots of normalized insertions mapped (NIM) and normalized reads mapped (NRM) scores generated by PIMMS counts. **(A)** Histogram of NRM = (total number of reads/length of gene in Kb) /total mapped read count/10^6^. This provides a number of reads mapped that is comparable between genes and experiments. **(B)** Histogram of NIM = (total unique insertions mapped/length of gene in Kb) /total insertions mapped/10^6^. This provides a number of insertions per gene that is comparable between genes and experiments. The distribution of NRM and NIM is skewed and highlights that many genes have a low NRM and NIM. The log transformation of these **(C,D)** provides an approximate normal distribution that allows comparison of NRM and NIM between experiments. The ratio and difference of these is compared in PIMMS compare module.

**FIGURE 6:**
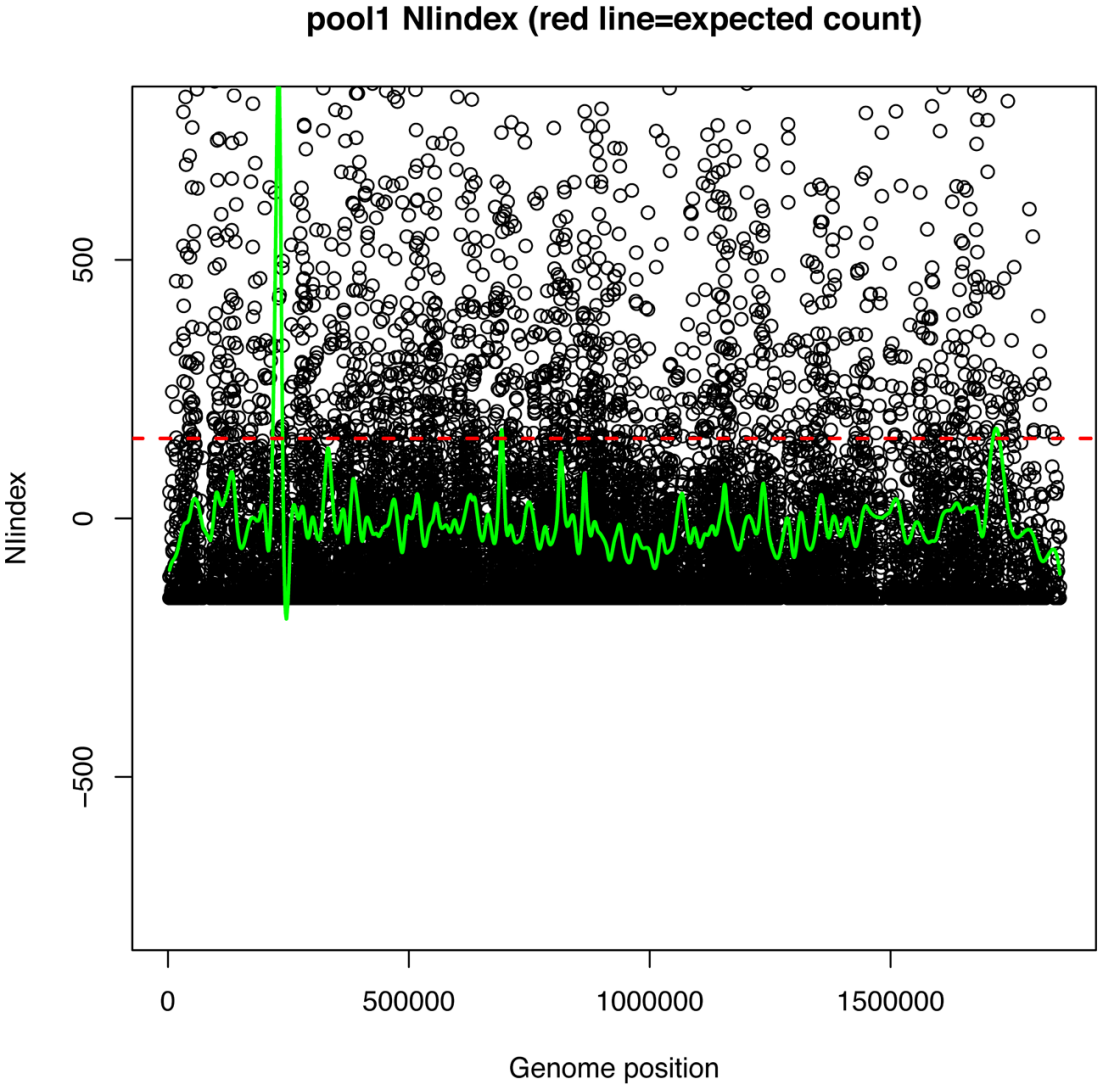
Example NIindex plots for single mutant pool. Circles represent the NIindex (observed count per position – expected count per position), where expected count per position determined as total mapped reads/unique mapped positions. If observed = expected NIindex would = 0. Green line is the smoothed summary of points. Red dotted line is the expected count per position.

**FIGURE 7:**
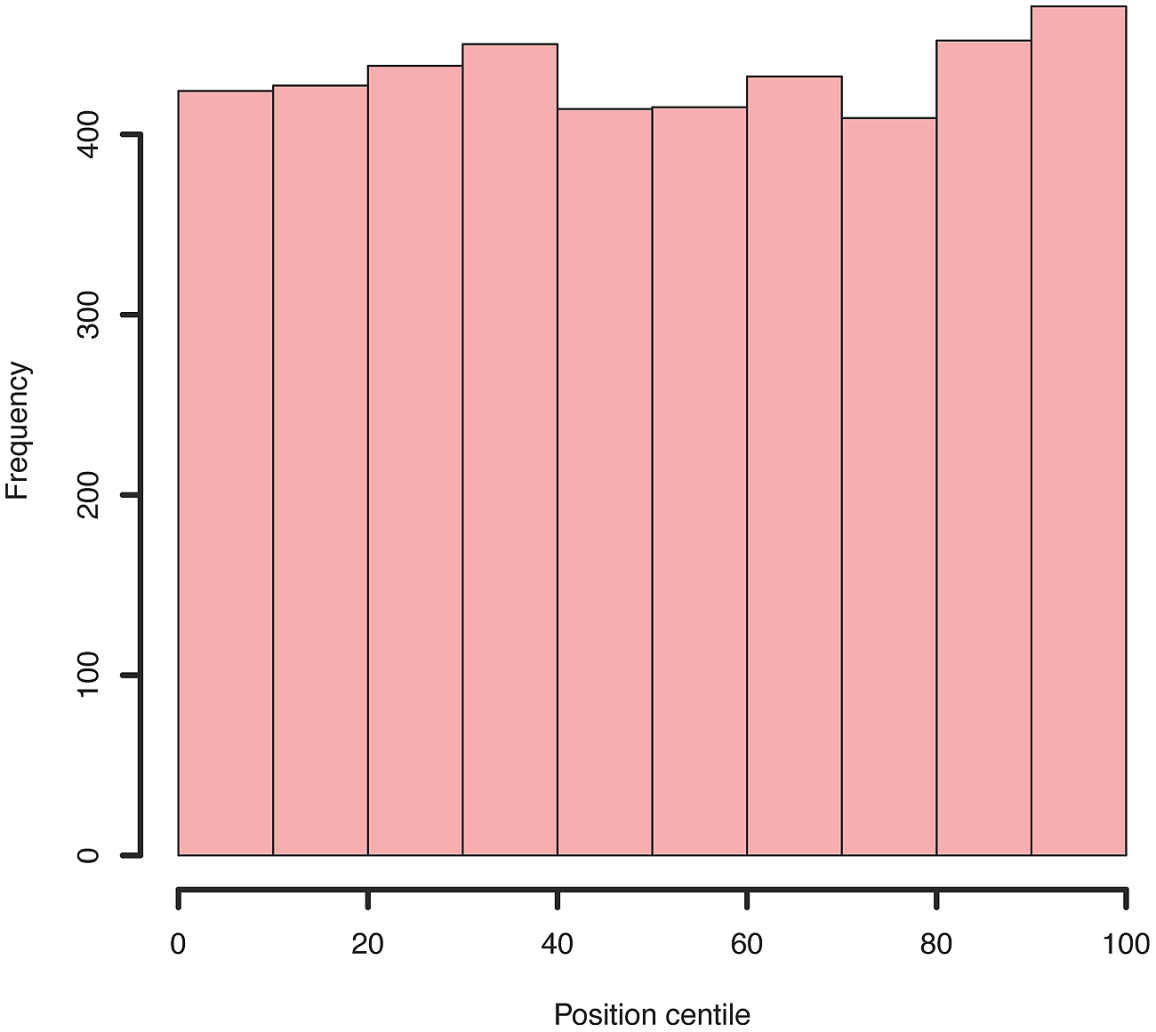
Example histogram of centile positions of all insertions within all genes. Whilst a very slight increase is seen toward the end of genes, this is clearly not a significant bias.

The PIMMS counts script requires a GFF file to match annotation to the insertion positions depth file generated by PIMMS process.sam. This is then used to generate tabulated output files (named × summary.table) of unique insertions, read depths at a position, normalized insertions per kb, the percentile position of the first and last insert within the coding sequence and normalized read values (NRM and NIM). NRM – Normalized Reads Mapped (total number of reads/length of gene in Kb)/(total mapped read count/10^6^) and NIM – Normalized Insertions Mapped (total unique insertions mapped/Length of gene in Kb)/(total insertions mapped/10^6^) provide an indication of the disruption of a given gene in comparison to others within the population and also takes into account the variability of the number of mapped sequence reads for each experiment. Summary figures of the distribution of NIM and NRM are generated (**Figure 5**) and plots of per position NIM together with a smoothed average plus expected insertion ratio allowing a simple visualization of “hot” or “cold” spots of insertions within the genome (**Figure 6**). In addition the distribution of the centile positions of insertions are generated (**Figure 7**). From our experience at a global level, there is little bias toward insertion in the start or end of protein coding genes within *Streptococcus uberis.* Underlying data for the generation of the plots are retained as text files so that additional plotting or investigation can be conducted by the user. To create a usable GFF2 file from an available EMBL file we recommend using seqret^2^.

### PIMMS Compare

The compare module allows processing of data obtained from phenotypic studies. Following use of the counts module, PIMMS compare, compares two pools termed “input” and “output” to identify common and unique mutation events between experimental conditions. Three output tables are produced (input only, output only, and shared positions). Tables consist of insertion position normalized observed and expected number of reads and associated gene information. For the shared positions an indication of the magnitude of deviation from an expected norm is determined. Within an experiment the natural logarithm (base *e*) transformed proportion ratio (the share of reads mapped at a location) approximates a normal distribution. Using the mean (shared mean) and standard deviation (shared sd) of this population, for each insertion the input/output proportion ratio (Proportion ratio) the Zscore is calculated as: Zscore = [(log(Proportion ratio)) – (shared mean)]/(shared sd). To provide an approximation of statistical importance, Zscores > SD equivalent to a *p*-value of 0.001, 0.01, 0.05 are flagged as |Zscore| > = 3.291, Flag = “^∗∗∗^” (∼*p*-value = 0.001), if 3.291 > |Zscore| > = 2.579, Flag = “^∗∗^” (∼*p*-value = 0.01), if 2.579 > |Zscore| > = 1.960, Flag = “^∗^” (∼*p*-value = 0.05) and if | Zscore| < 1.960, Flag = “”.

## Analysis of Existing Data using PIMMS

**FIGURE 8:**
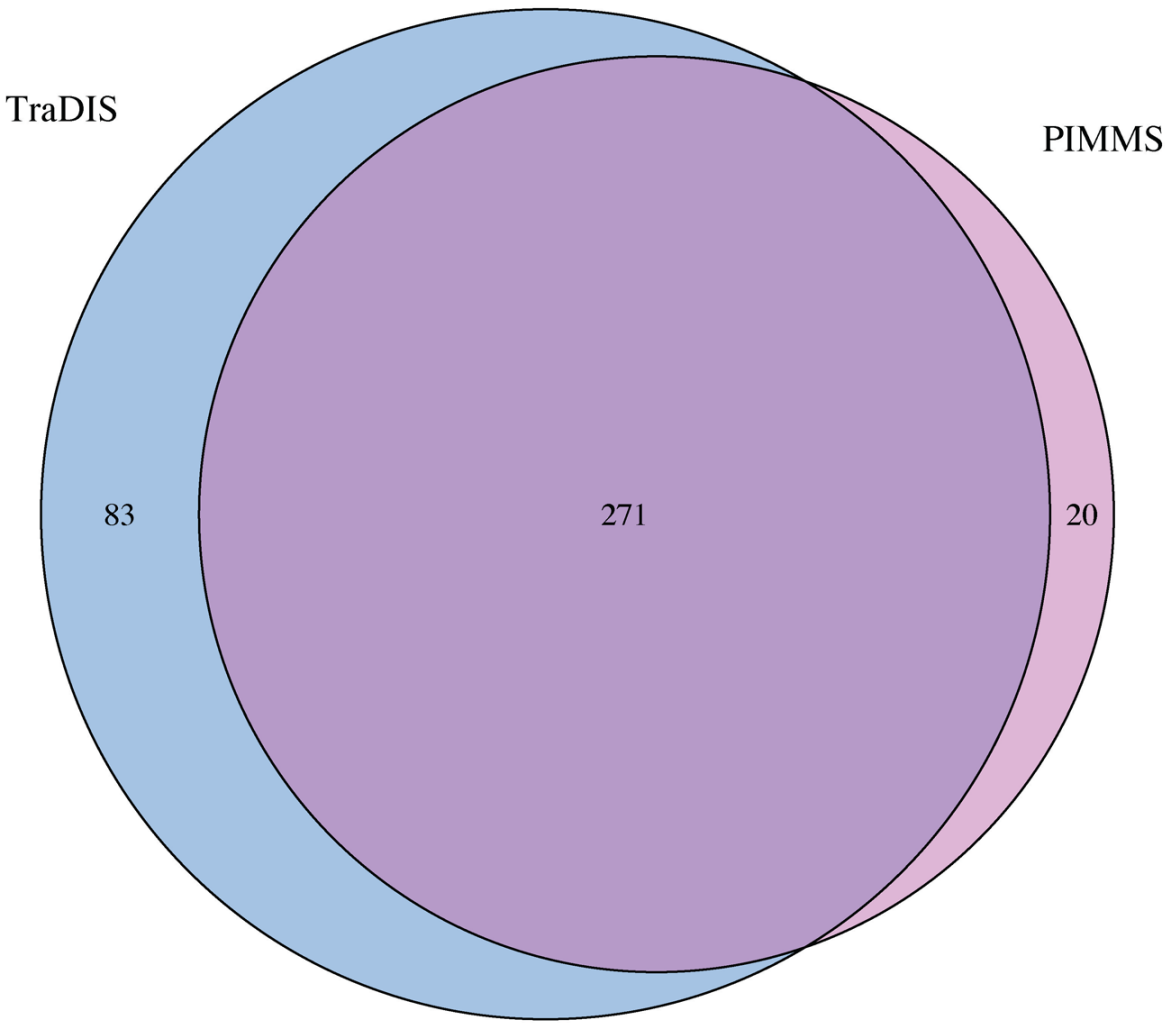
Overlap between identified essential genes detected by TraDIS (see Langridge et al., 2009 for data) and PIMMS. Genes were classified as essential if log_2_-LR < -2 (TraDIS) or NIM < 2 (PIMMS).

**FIGURE 9:**
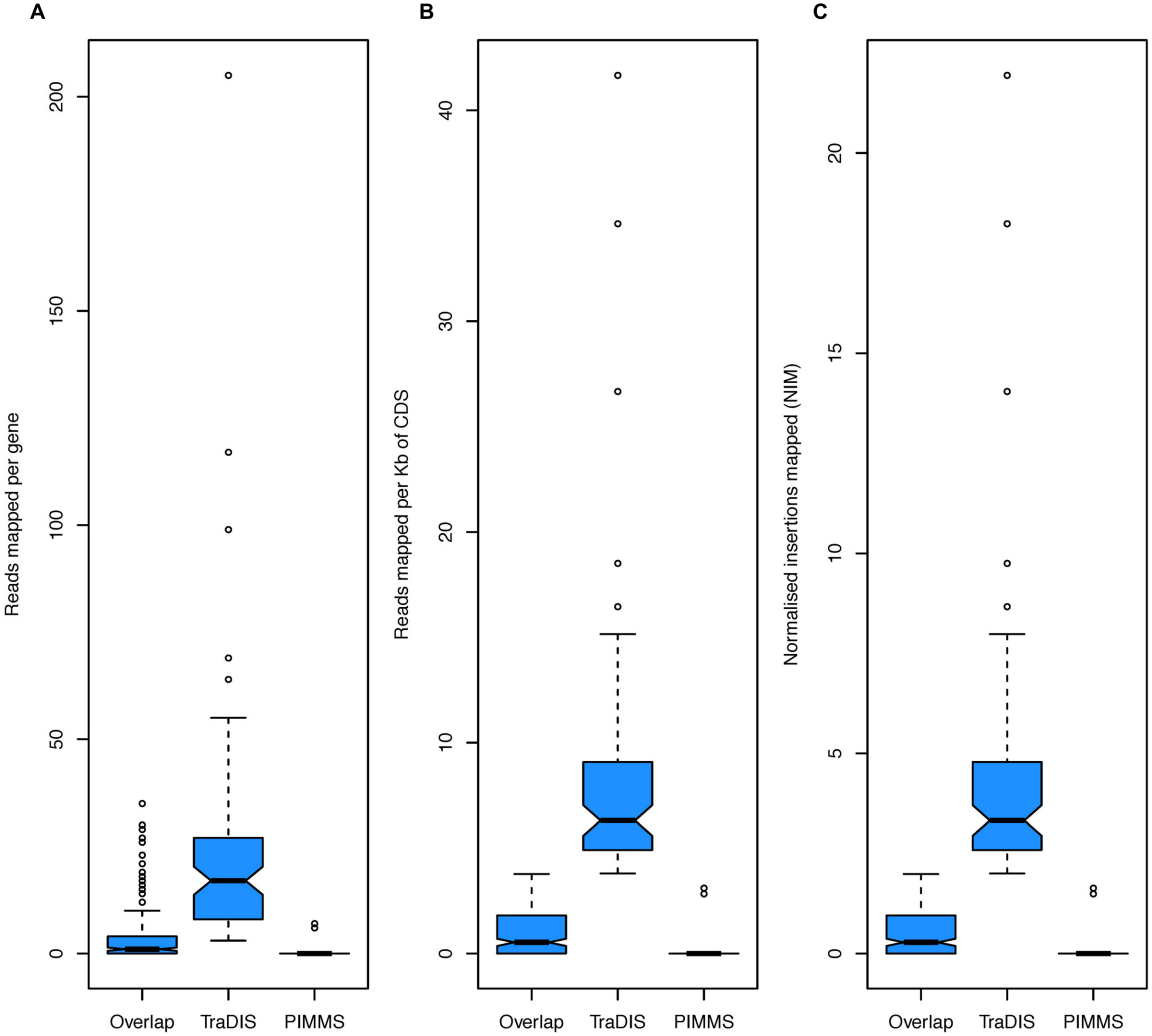
Boxplots of **(A)** total reads mapped to gene, **(B)** total reads mapped normalized by read length of gene, and **(C)** NIM. In each plot the group “Overlap” corresponds to genes identified as essential by TraDIS and PIMMS (271 genes), “TraDIS” corresponds to genes identified by TraDIS only (83 genes) and “PIMMS” corresponds to genes identified by PIMMS only (20 genes).

To compare the performance and utility of PIMMS, sequence reads generated as part of the study of essential genes in *Salmonella typhi* (Langridge et al., 2009) were analyzed. The data set ERR004088 available from the sequence read archive^3^ comprises 14,201,779 single end reads. Using the TraDIS method Langridge et al. (2009) reported mapping of 370,000 insertions at a mean inter-insertion distance of 13 bp (Langridge et al., 2009). Using PIMMS with the default mapper (BWA –mem) and fragment retention sizes of –minimum 20 –maximum 50. Resulted in 8,580,710 reads matching the transposon motif TAAGAGACAG. Following filtering for alignment quality (0 mismatches and alignment score of >20 [PIMMS -m process.sam -c N -mis 0 -a 20]) 1,898,673 reads confidently mapped in a total of 321,514 unique insertion positions with mean inter-insertion distance of 14.9 bp comparable to the original TraDIS experiment. Using the Langridge et al. (2009) definition of essentiality (log_2_-LR < -2) or PIMMS NIM <2, showed largely consistent results between PIMMS and TraDIS (**Figure 8**). The 83 genes identified as essential by TraDIS only may possibly represent misalignment during the PIMMS procedure, as the sequence length from Langridge et al. (2009)is short (50 bp single end) compared to the standard 2 × 250 bp paired end sequencing of the PIMMS approach. To overcome this the PIMMS alignment stringency had to be reduced from our recommended 50 to 20. This may lead to some short reads being inappropriately mapped, however, many of the genes identified as essential by Langridge et al. (2009) show high numbers of raw or normalized insertions suggesting non-essentiality (**Figure 9**). Twenty genes are identified by PIMMS only. Fourteen of these are transfer-RNAs possibly not considered by Langridge et al. (2009) Two *S. typhi* genes, t0860, and t2722 encode hypothetical proteins. Others, t3477 (50S ribosomal subunit protein L1), t3650 (ATP synthase subunit B), t0803 (his operon leader peptide), and t0095 (survival protein SurA precursor) encoded proteins of known function. Evidence for these being truly essential can only be confirmed by experimental validation, but a homolog of SurA is essential for growth in *Escherichia coli* (Tormo et al., 1990) suggesting it may hold a similar role in *S. Typhi* and many of the ribosomal subunit proteins are detected as essential by both TraDIS and PIMMS. This suggests that genes identified by PIMMS are worthy of inclusion as possible essential genes.

## Conclusion

Currently the major software available for analysis of transposon mediated mutagenesis data and identification of essential genes is ESSENTIALS (Zomer et al., 2012). However, use of this web-based tool may be limited if data cannot be transferred and stored on-line. Whilst a standalone version of ESSENTIALS can be obtained from the developer, this version requires modifications which may be beyond the abilities of a novice user. ESSENTIALS uses FASTX toolkit to identify and trim transposon sequence and PASS (Campagna et al., 2009) to align trimmed sequences. Essential genes are then identified by comparison of mapped counts using EdgeR (Robinson et al., 2010). The power of EdgeR when multiple replicates are available is well established, however, where reduced replicates are available or where greater user control of choice of aligner and parameters of aligner and^4^ mapping stringency are required PIMMS provides a compelling alternative.

In comparison to TraDIS, PIMMS is a truly pragmatic choice. Whilst the results are largely comparable, TraDIS requires complex preparation of sequence data including PCR library preparation with custom Illumina primers. PIMMS relies on generation of libraries using standard protocols following inverse PCR or even restriction fragment digestion (Blanchard et al., unpublished). In addition the PIMMS processing pipeline is quick, taking less than 10 min on a desktop computer (i7-3820 CPU @ 3.60 GHz, running Ubuntu 14.04) to complete all step of transposon matching, mapping and results processing. Therefore, PIMMS analysis pipeline provides a convenient, robust, and importantly reproducible toolkit to explore and prioritize output from vast amounts of sequencing data required to map transposon generated insertions within a population, without the need for complex data manipulation by multiple tools.

## Conflict of Interest Statement

Richard D. Emes is Speciality Chief Editor of Frontiers in Bioinformatics and Computational Biology. The other authors declare that the research was conducted in the absence of any commercial or financial relationships that could be construed as a potential conflict of interest.

## Footnotes

1 http://samtools.github.io/hts-specs/

2 emboss.sourceforge.net/apps/release/6.6/emboss/apps/seqret.html

3 http://www.ncbi.nlm.nih.gov/sra/?term=ERR004088

4 http://hannonlab.cshl.edu/fastx_toolkit/
